# The migration speed of nucleolar precursor bodies in pronuclei affects in vitro fertilization‐derived human embryo ploidy status and live birth

**DOI:** 10.1002/rmb2.12497

**Published:** 2023-01-20

**Authors:** Taketo Inoue, Sayumi Taguchi, Mikiko Uemura, Yoshiko Tsujimoto, Kana Kokunai, Kayoe Ikawa, Yoshiki Yamashita

**Affiliations:** ^1^ Umeda Fertility Clinic Osaka Japan; ^2^ Department of Emergency, Disaster and Critical Care Medicine Hyogo Medical University Nishinomiya Japan; ^3^ Department of Rehabilitation, Faculty of Health Science Kansai University of Welfare Sciences Osaka Japan

**Keywords:** embryo development, live birth, nucleolus precursor body, pronuclei, zygote

## Abstract

**Purpose:**

To study the relationship between clinical outcomes after assisted reproduction and the migration speed of nucleolus precursor bodies (NPBs) in male and female pronuclei (mPN; fPN).

**Methods:**

NPB migration speed, embryo ploidy status, and live birth (LB) were retrospectively analyzed in IVF‐derived zygotes. The central coordinates of the mPN, fPN, and NPBs were noted at multiple timepoints. The migration distance of NPBs between two sequential images was measured to calculate NPB migration speed.

**Results:**

The NPB migration speeds in mPN and fPN were significantly faster in euploid zygotes than in aneuploid zygotes. In multivariate logistic analysis, NPB migration speed in mPN and the female age were associated with euploidy. The NPB migration speeds in mPN and fPN were also significantly faster in zygotes that led to LB than in zygotes that led to no pregnancy. In a receiver operating characteristic curve analysis of LB by NPB migration speed in mPN, the cut‐off value was 3.74 μm/h (AUC: 0.825, 95%CI: 0.688–0.963). When the zygotes were categorized by this cut‐off value, there were significantly more LBs in zygotes with migration speed ≥ the cut‐off (78.9% vs. 21.1%).

**Conclusions:**

Zygotes with quickly migrating NPBs demonstrated the developmental potential to become a baby.

## 1 INTRODUCTION

Nuclear bodies, RNA‐ and protein‐rich, membraneless organelles, play a crucial role in gene regulation.^1^ Nucleoli in mammalian oocytes and zygotes, which are referred to as nucleolus‐like bodies and nucleolus‐precursor bodies (NPBs), are compact, ribosome‐biosynthesis‐inactive structures, and are distinct morphologically from the nucleoli in somatic cells.^2^, ^3^ During the oocyte maturation, nucleolus‐like bodies are found in fully grown, germinal vesicle stage oocytes and then disappear. After meiotic division and fertilization, NPBs are formed in the male pronuclei (mPN) and female pronuclei (fPN) of the zygote and participate in genome remodeling and organization during embryonic development.^3^, ^4^, ^5^ NPBs are essential for embryonic development after fertilization and may be actively involved in establishing centromeric chromatin.^4^, ^6^

NPBs are surrounded by pericentromeric and centromeric heterochromatin in a ring‐like shape.^5^ NPB position correlates with the distribution of chromatin, and NPB repositioning reflects a global redistribution and clustering of chromatin at the interpronuclear interface.^7^, ^8^ The parental genomes cluster with nucleoli in each pronucleus within a human zygote, and clustering is required for the reliable unification of the parental genomes after fertilization.^8^ During syngamy, NPBs move extensively and characteristic NPB patterns change.^9^, ^10^ The NPB migration speed in intracytoplasmic sperm injection (ICSI)‐derived human zygotes predicts the competence of the embryo to develop to the blastocyst stage and result in a live birth (LB).^11^

There are key differences between in vitro fertilization (IVF) and ICSI during fertilization process. ICSI bypasses several biological barriers that naturally select the gametes best suited for optimal embryonic and fetal development, such as natural spermatozoa selection.^12^ During ICSI, the sperm plasma membrane and acrosome, which contains hydrolyzing enzymes, are introduced into the ooplasm.^13^ Oocyte activation, as indicated by Ca^2+^ oscillations, after fertilization differs in IVF and in ICSI.^14^ DNA replication in the mPN, but not in the fPN, is delayed after the ICSI procedure.^15^ Whether these differences in the fertilization process affect NPB migration speed or the ability of NPB migration speed to predict LB was unclear after our previous study.^11^ Therefore, in this study, we investigated the relationship between NPB migration speed, ploidy status, and clinical outcomes after assisted reproductive technology (ART) in IVF‐derived zygotes to determine if differences in the fertilization procedure affect NPB migration and the clarify the relationship between NPB migration, ploidy, and LB in IVF‐derived zygotes.

## 2 MATERIALS AND METHODS

### 2.1 Patients

The relationship between NPB migration and ploidy status in IVF‐derived zygotes was retrospectively analyzed in patients with recurrent ART failure (euploid: *N* = 18; aneuploid: *N* = 19; 220 NPBs). The mean (±SD) age of the women who participated was 38.5 ± 4.2 years when ovum pickup (OPU) was performed (range 29–43 years). Data were obtained from 24 IVF cycles between February 2020 and October 2021.

Next, the relationship between NPB migration speed and clinical outcomes was retrospectively analyzed using records obtained from patients who underwent IVF with vitrified‐warmed, single IVF‐derived blastocysts without preimplantation genetic testing for aneuploidy (PGT‐A) with transfer cycles between October 2019 and September 2021. The mean age of these women was 33.0 ± 3.2 years (range 28–40 years) when OPU was performed. A freeze‐all strategy was used in these patients to reduce the risk of ovarian hyperstimulation syndrome during the ovarian stimulation cycle. The data from 19 patients with LB and 19 patients with no pregnancy (gestational sac [GS] was negative) were retrieved, and 225 NPBs were analyzed.

Third, the relationship between NPB migration speed and miscarriage was retrospectively analyzed using records obtained from patients who underwent IVF with vitrified‐warmed, single IVF‐derived blastocysts/embryos without PGT‐A with transfer cycles between November 2019 and July 2022. The mean age of these women was 36.0 ± 4.7 years (range 25–42 years) when OPU was performed. The data from patients with miscarriages (fetal‐heart‐movement [FHM]+/LB−, *N* = 8; GS+/FHM−, *N* = 14) or biochemical pregnancy (human chorionic gonadotropin [hCG]+/GS−; *N* = 14) were retrieved, and 108 NPBs in mPN were analyzed. The FHM+/LB− miscarriages comprised 7 spontaneous abortions at 7–9 weeks and 1 intrauterine fetal death at 23 weeks.

Additionally, the relationship between NPB migration speed and clinical outcome after ART in female patients ≥40 years old was retrospectively analyzed using records obtained from patients who underwent IVF with vitrified‐warmed, single IVF‐derived blastocysts/embryos without PGT‐A with transfer cycles between February 2020 and July 2022. The mean age of these women was 41.0 ± 1.0 years (range 40–43 years) when OPU was performed. The data from 5 patients with live birth/ongoing pregnancy (>27 weeks) (LB/OP), 6 patients with miscarriage, and 5 patients with no pregnancy were retrieved, and 48 NPBs in mPN were analyzed. The 6 patients with miscarriage were also included in the miscarriages group described above because few patients met these criteria.

After patients with LB, no pregnancy, miscarriage, or recurrent ART failure were identified for the study, archived time‐lapse videos from incubation after IVF were retrieved, and NPB migration speed was analyzed. The retrospective analyses were performed with the patients' identities masked. Sample size was calculated a priori using an effect size of *d* = 0.949 based on data of LB/OP in our previous study,^11^ *α* error probability = 0.05, power (1−*β* error probability) = 0.8, and allocation ratio (N2/N1) = 1. This study was approved by our Institutional Review Board, and informed consent was obtained from all patients.

### 2.2 Controlled ovarian stimulation/oocyte retrieval

Controlled ovarian stimulation was performed using a long, short, or gonadotropin‐releasing hormone (GnRH) antagonist protocol depending on the patient as described previously.^16^, ^17^, ^18^ A GnRH analogue acetate (Fuji Pharma Co., Tokyo, Japan), human menopausal gonadotropin (ASKA Pharmaceutical Co., Tokyo, Japan), and a GnRH antagonist (Cetrotide; Merck KGaA, Darmstadt, Germany) were administered as dictated in these established protocols. When at least 2 follicles reached 18–20 mm in diameter (as determined by transvaginal ultrasonography), 5000 IU of hCG (Fuji Pharma Co.) was administered. Oocyte retrieval was performed 36 h after hCG injection. The cumulus‐oocyte complexes were placed into 4‐well dishes (Thermo Fisher Scientific, Waltham, MA, USA) containing HFF99 medium (Fuso Pharmaceutical Industries, Osaka, Japan) with 10% serum protein substitute (Kitazato Corporation, Shizuoka, Japan) and cultured for 2 to 3 h until insemination.

### 2.3 In vitro fertilization and time‐lapse microscopy

Ejaculated sperm were collected and incubated at room temperature for at least 30 min. Semen samples were analyzed using a Makler Counting Chamber (Sefi Medical Instruments, Haifa, Israel) and were overlaid onto 90% ISolate (Fujifilm Irvine Scientific, Santa Ana, CA, USA) and then centrifuged at 1800 revolutions per minute (rpm) for 15 min. The supernatant was removed, and 0.5 to 1 ml of Gems Fertilization Medium (Gems: Genea Biomedx Sydney, NSW, Australia) was added to the pellet. The swim‐up technique was used for 30 min in an incubator (CO_2_, 6%; O_2_, 5% at 25°C and 100% humidity) to select spermatozoa. After swim‐up spermatozoa were obtained, approximately 10 × 10^4^ swim‐up sperm per ml were added to a center‐well organ culture dish (Falcon, Corning Life Sciences, Tewksbury, MA, USA) containing <5 cumulus‐oocyte complexes in 1 ml of Gems Fertilization Medium. After 4.5 h, oocytes were freed from cumulus cells using pipette.

After confirming that polar bodies were present, inseminated oocytes were placed into individual wells in a Geri® dish (Genea Biomedx) in a time‐lapse incubator (Geri+; Genea Biomedx) and cultured (CO_2_, 6%; O_2_, 5% at 37°C and 76.5% humidity) in an 80‐μl drop of Geri medium covered with 4.0 ml of light mineral oil (Oil for Embryo Culture; Fujifilm Irvine Scientific) for 7 days. The blastocysts were scored according to the criteria of Gardner and Schoolcraft.^19^

Time‐lapse microscopy is suitable to assess dynamic morphological processes and is an established tool for non‐invasive dynamic observation of human embryos. Images were recorded every 5 min. The timing of PN appearance (tPNa), the timing of PN disappearance (tPNf), and the timing of the divisions resulting in 2–8 cells (t2–t8), morula formation (tM), and blastocyst formation (tB; cavitation) from the completion of insemination were annotated.

### 2.4 Measurement of the migration speed of nucleolus precursor bodies

**FIGURE 1:**
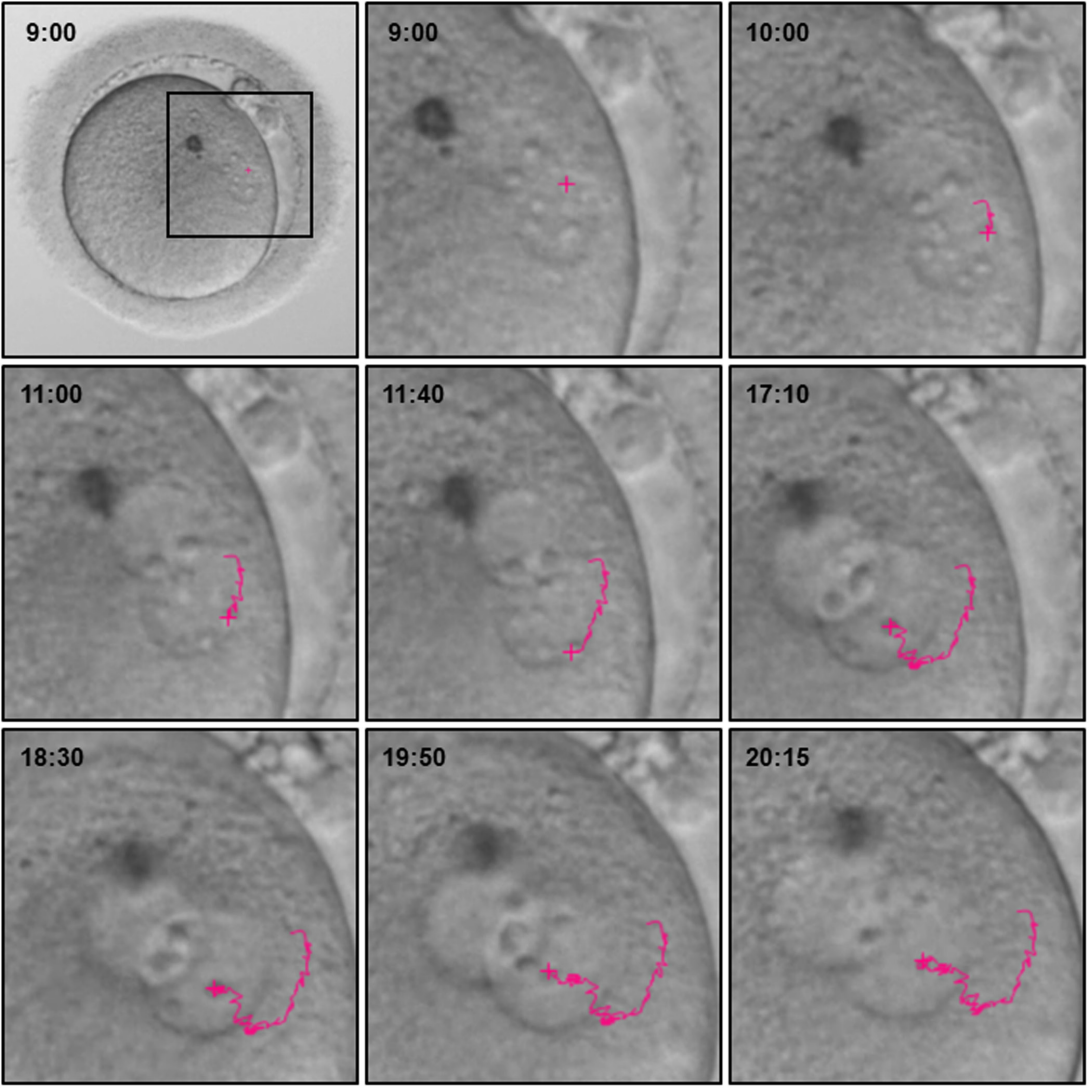
NPB tracking. Time: From the completion of insemination.

Measurement of the migration speed of NPBs was performed using time‐lapse microscopy as described previously.^11^ When selecting NPBs for analysis, first, we selected NPBs with as long of an appearance period as possible and an appearance period of at least 4–5 h to reduce variability when measuring average speed. Second, we selected NPBs that were moving quickly throughout the observation period. The mPN and fPN were identified based on the location where they first appeared in the zygote. The fPNs appear just below the polar bodies. The central coordinates of the mPN, fPN, and 2–3 NPBs/PN were measured using Kinovea motion capture software (Patreon, San Francisco, CA, USA) and were confirmed or revised in every image (Figure 1). The migration distance of NPBs between two sequential images was calculated relative to the central coordinates of the PN. Thereafter, the migration speed of the NPBs was calculated. The mean frames analyzed were 126.5 ± 38.4. The following equations were applied:

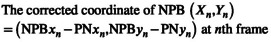




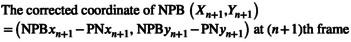

$$\text{The migration length of}\;\mathrm{NPB}\;\left(\mathrm{\mu m}\right)=\sqrt{{\left(X_{n+1}-X_{n}\right)}^{2}+{\left(Y_{n+1}-Y_{n}\right)}^{2}}$$

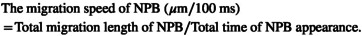

Zygotes with smooth endoplasmic reticulum clustering, numerous cytoplasmic granules, numerous viscosities, overlapping PN, or bull's‐eye PN were excluded from the analysis.

### 2.5 Cryopreservation, warming, and blastocyst/embryo transfer

Blastocysts and embryos were vitrified and warmed using the Cryotop technique, as previously described.^20^ A single, vitrified‐warmed blastocyst or embryo was transferred to the uterus of each patient. Luteal support for vitrified‐warmed blastocyst/embryo transfer (VBT/VET) cycles was performed as previously described.^11^ After the initiation of menstruation, 0.72 mg transdermal estradiol patches (Estrana Tape, Hisamitsu Pharmaceutical Co., Tokyo, Japan) were applied to the abdomen for 10 weeks. Two 200 mg progesterone vaginal suppositories (Utrogestan vaginal capsules 200 mg, Fuji Pharma. Co.) were administrated 2 times per day beginning when the endometrial thickness was at least 7.0 mm. Beginning on the day of VBT/VET, the women were injected with 125 mg of 17α‐hydroxyprogesterone caproate (progesterone depot intramuscular injection; Fuji Pharma. Co.) once every 5 days for the first 8 weeks of pregnancy. Pregnancy and ongoing pregnancy were determined by the ultrasonographic observation of a GS and observation of a fetus. A LB was defined to be the complete expulsion or extraction from the mother of a baby that after separation breathed or showed any other evidence of life, such as beating of the heart, pulsation of the umbilical cord, or definite movement of the voluntary muscles, independent of whether the umbilical cord or placenta was attached and irrespective of the duration of the pregnancy.^21^

### 2.6 Biopsy of blastocysts and chromosome analysis

The biopsy of blastocyst and the storage/transportation of the biopsy sample were performed according to instructions from the genetic testing agency (Igenomix, Valencia, Spain). Assisted hatching was performed on all blastocysts before biopsy with a hole of approximately 20 μm using the OCTAX Laser & Imaging system (OCTAX microscience GmbH, Bruckberg, Germany). Biopsy of the blastocysts by the mechanical “flicking” method was performed in 10‐μl droplets of modified human tubal fluid medium (mHTF, Kitazato Corporation) with HEPES (N‐2‐hydroxyethylpiperazine‐N2‐ethane sulfonic acid) containing 10% serum protein substitute after trophectoderm (TE) herniation. Approximately 5–10 TE cells were sent to an outside laboratory for PGT‐A. The TE sample was rinsed with 5‐μl droplets of 1% polyvinylpyrrolidone in phosphate‐buffered saline 3 times and transferred to a PCR tube containing 2.5 μl of loading solution (phosphate‐buffered saline) under the microscope. The sample was stored at −20°C prior to transportation.

Cell lysis of the TE samples, DNA extraction, whole‐genome amplification (WGA), and DNA barcoding were performed using the Ion ReproSeq PGS Kit (Thermo Fisher Scientific). WGA samples were evaluated by loading 1.5 ml of the amplified products onto an agarose gel (Lonza, Basel, Switzerland), and were pooled in batches of 96 samples. Libraries were quantified using the Qubit High Sensitivity dsDNA Kit (Life Technologies, Carlsbad, CA, USA), diluted, and loaded onto Ion Chef (Thermo Fisher Scientific) for automated template preparation and chip loading. Sequencing of the chips was done using S5 sequencer (Thermo Fisher Scientific). The data obtained were analyzed using Ion Reporter software. Mosaicism was determined by the proprietary algorithm of the genetic testing agency (Igenomix), and results were classified as euploid when mosaicism was detected in <30% of cells and as aneuploid when mosaicism was detected in >30% of cells. Aneuploidy was classified as monosomy (*N* = 6), trisomy (*N* = 5), or complex abnormal (monosomy + trisomy; *N* = 8).

### 2.7 Statistical analysis

All data are presented as mean ± standard deviation. Normality was tested with Shapiro–Wilk normality tests, and analysis of variance was performed by *F*‐test. Consequently, Student's *t*‐test, Welch's *t*‐test, or Mann–Whitney *U* test was used to compare average differences between 2 groups. Spearman rank‐order correlation coefficient (Spearman's correlation, *γs*) was used to assess the correlation between 2 factors. Tukey–Kramer's post hoc test was carried out to evaluate multiple comparisons after the analysis for homogeneity of variance was performed by Bartlett test.

The relationship between ploidy status and 35 parameters was analyzed using univariate logistic analysis. Subsequently, multivariate logistic analysis was conducted using factors with *p* < 0.15 in the univariate logistic analysis. To avoid multicollinearity problems, parameters with a variance inflation factor ≥5 were removed one by one until values of all variance inflation factors became <5. The confounding factors were omitted in order from the factor with the largest *p* value after multiple logistic regression analysis. Statistical analysis was then repeatedly performed until *p* values of all confounding factors became <0.05 (backward stepwise selection).

Receiver‐operating‐characteristic (ROC) curve analysis was used to calculate the cut‐off values for ploidy status and LB; mNPB and fNPB migration speed as predictors of euploidy and LB were evaluated using a bootstrap test for 2 correlated ROC curves. Inner cell mass (ICM) and TE grades were compared using the chi‐square test and residual analysis. Ploidy status categorized by cut‐off values and LB categorized by a cut‐off value were evaluated using Fisher's exact probability test. A probability level of *p* < 0.05 was considered statistically significant. The statistical analysis was performed using EZR software.^22^ An a priori sample size calculation was performed using G*Power,^23^ and effect size was calculated by referring to data of our previous study.^11^ To determine if the migration speed of the NPBs was assessed in enough PN to make the study meaningful, a post hoc power analysis was performed using G*Power, and power (1−*β*) was judged according to the criteria of Cohen (1−*β* > 0.8 indicates adequate power).^24^, ^25^

## 3 RESULTS

### 3.1 The migration speed of NPBs in the mPN and fPN is correlated with ploidy status

**FIGURE 2:**
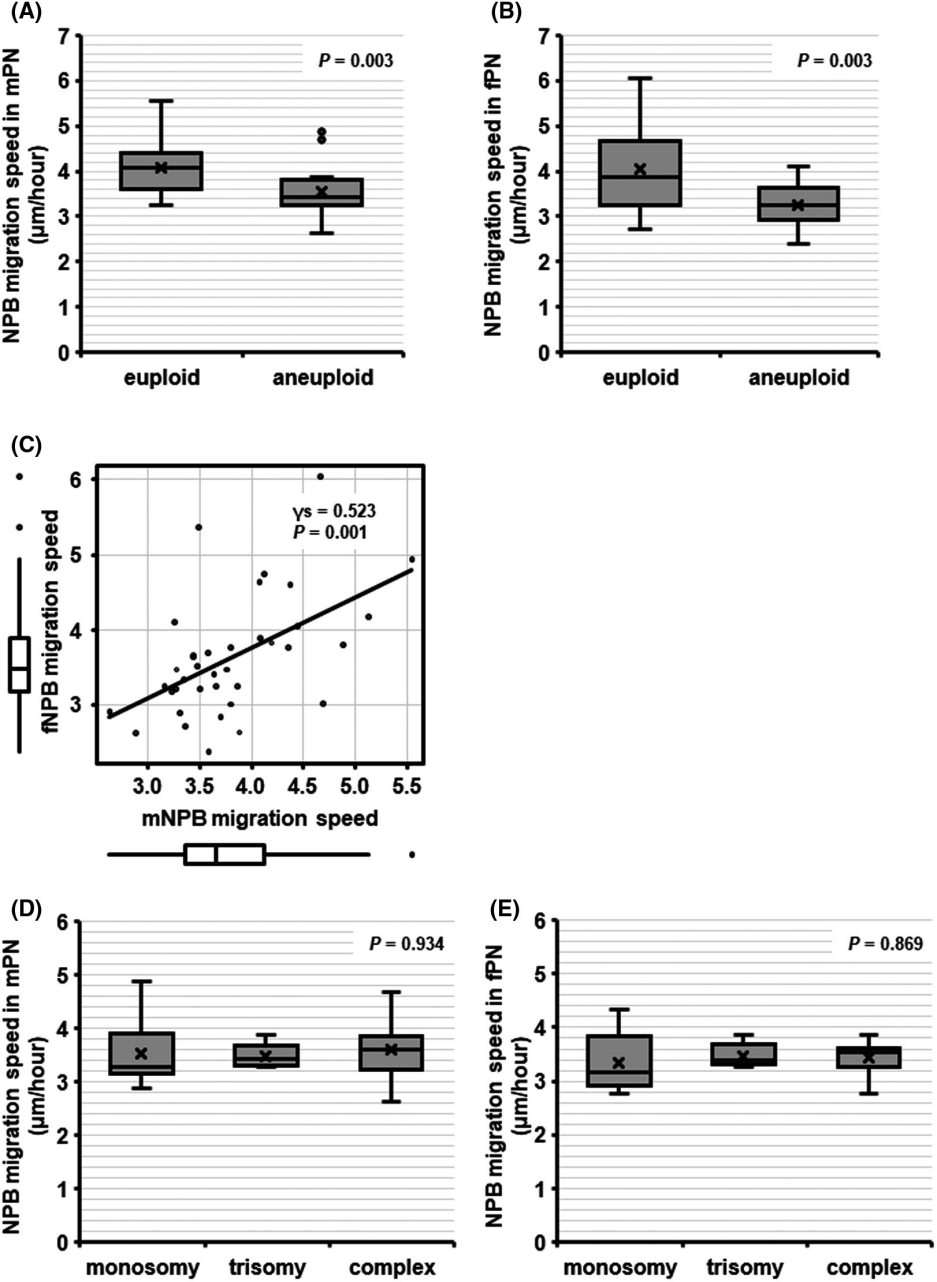
The relationship between migration speed of NPBs and human embryo ploidy status (euploid: *N* = 18; aneuploid: *N* = 19; 220 NPBs). The migration speed of NPBs in (A) mPN and (B) fPN. The migration of both mNPBs and fNPBs in euploid embryos was significantly faster than in aneuploid embryos (mNPBs: 4.08 ± 0.61 vs. 3.54 ± 0.54 μm/h; fNPBs: 4.03 ± 0.89 vs. 3.26 ± 0.45 μm/h). (C) the relationship between the migration speed of mNPBs and the migration speed of fNPBs. The migration speed of NPBs in (D) mPN and (E) fPN in aneuploid embryos. The migration speeds of mNPB and fNPB in aneuploid embryos were similar among embryos with different types of aneuploidy (monosomy vs. trisomy vs. complex abnormalities; mNPBs: 3.52 ± 0.70 vs. 3.48 ± 0.24 vs. 3.60 ± 0.59 μm/h; fNPB: 3.35 ± 0.58 vs. 3.47 ± 0.24 vs. 3.44 ± 0.33 μm/h). fNPB, nucleolus precursor bodies in female pronuclei (fPN); mNPB, nucleolus precursor bodies in male pronuclei (mPN); γs, spearman correlation.

To investigate the relationship between the migration speed of NPBs and ploidy status, we tracked NPBs using time‐lapse microscopy in IVF‐derived zygotes from patients with recurrent ART failure. Blastocyst quality, as indicated by ICM and TE grades,^19^ was not different between the euploid and aneuploid embryos (Table 1). The migration speed was quantitated for 220 NPBs—111 NPBs in mPN (mNPBs) and 109 NPBs in fPN (fNPBs)—in 37 zygotes (3.00 ± 0.00 mNPBs/zygote and 2.95 ± 0.23 fNPBs/zygote). The migration of both mNPBs and fNPBs in euploid embryos was significantly faster than in aneuploid embryos (mNPBs: 4.08 ± 0.61 vs. 3.54 ± 0.54 μm/h, *p* = 0.003, 1−*β* = 0.776; fNPBs: 4.03 ± 0.89 vs. 3.26 ± 0.45 μm/h, *p* = 0.003, 1−*β* = 0.903) (Figure 2A,B). The migration speed of mNPBs was positively correlated with that of fNPBs (*γs* = 0.523, *p* = 0.001) (Figure 2C). The migration speeds of mNPB and fNPB in aneuploid embryos were similar among embryos with different types of aneuploidy (monosomy vs. trisomy vs. complex abnormalities; mNPBs: 3.52 ± 0.70 vs. 3.48 ± 0.24 vs. 3.60 ± 0.59 μm/h, *p* = 0.934; fNPB: 3.35 ± 0.58 vs. 3.47 ± 0.24 vs. 3.44 ± 0.33 μm/h, *p* = 0.869) (Figure 2D,E).

**TABLE 1:** Characteristics of blastocysts with preimplantation genetic testing for aneuploidy

	Euploid	Aneuploid	*p* value
No. of blastocysts	18 (48.6%)	19 (51.4%)	
Controlled ovarian stimulation			
Long protocol	2 (11.1%)	1 (5.3%)	0.705
Short protocol	7 (38.9%)	7 (36.8%)	
Antagonist protocol	9 (50.0%)	11 (57.9%)	
Inner cell mass grade, *n* (%)			
Grade A	0 (0%)	0 (0%)	0.317
Grade B	9 (50.0%)	12 (63.2%)	
Grade C	9 (50.0%)	7 (36.8%)	
Trophectoderm grade, *n* (%)			
Grade A	2 (11.1%)	1 (5.3%)	0.182
Grade B	7 (38.9%)	3 (15.8%)	
Grade C	9 (50.0%)	15 (78.9%)	

To confirm the correlation of known morphokinetic parameters with ploidy status, we quantitated 35 factors including the age of the patient providing the oocytes and the age of the patient providing the sperm, time of cleavage, cleavage intervals from completion of insemination to blastulation, and ICM/TE grades. Ploidy status was correlated with the migration speed of mNPBs and fNPBs in an univariate logistic analysis (Table 2). In multivariate logistic analysis, only the migration speed of the mNPBs (odds ratio [OR], 10.2; 95% confidence interval [CI], 1.90–54.90; *p* = 0.007) and age of the patient providing the oocyte (OR, 0.8; 95% CI, 0.64–0.98; *p* = 0.031) were associated with ploidy status (Table 2).

**TABLE 2:** The relationship between NPB migration speed and ploidy

	Logistic analysis					Comparison between two groups^a^		
	*β*	SE *β*	Odds ratio	95% CI	*p* value	Euploid	Aneuploid	*p* value
Univariate logistic analysis								
Female (oocyte provider) age (years)	−0.127	0.085	0.881	0.745–1.040	0.139	37.4 ± 4.4	39.5 ± 3.9	0.092
Male (sperm provider) age (years)	−0.056	0.069	0.946	0.827–1.080	0.417	37.7 ± 6.23	39.0 ± 3.6	0.434
Migration speed of mNPBs (μm/h)	1.810	0.763	6.110	1.370–27.300	0.018	4.1 ± 0.6	3.5 ± 0.5	0.003
Migration speed of fNPBs (μm/h)	1.897	0.737	6.660	1.570–28.200	0.010	4.0 ± 0.9	3.3 ± 0.5	0.003
tPNa (h)	−0.213	0.261	0.808	0.485–1.350	0.413	8.5 ± 1.2	8.9 ± 1.4	0.423
tPNf (h)	−0.042	0.122	0.959	0.756–1.220	0.732	23.7 ± 2.4	24.0 ± 3.1	0.740
t2 (h)	−0.021	0.118	0.979	0.776–1.230	0.859	26.1 ± 2.5	26.3 ± 3.2	0.864
t3 (h)	−0.121	0.094	0.887	0.737–1.070	0.204	35.5 ± 3.3	37.0 ± 4.0	0.206
t4 (h)	−0.077	0.102	0.926	0.759–1.130	0.449	37.7 ± 2.9	38.5 ± 3.8	0.460
t5 (h)	−0.028	0.056	0.973	0.871–1.090	0.622	49.2 ± 5.3	50.1 ± 6.6	0.633
t6 (h)	−0.071	0.068	0.931	0.815–1.060	0.292	51.0 ± 4.5	52.8 ± 5.6	0.299
t7 (h)	−0.039	0.061	0.962	0.854–1.080	0.524	53.3 ± 5.0	54.5 ± 6.1	0.536
t8 (h)	−0.038	0.054	0.963	0.866–1.070	0.487	55.4 ± 6.0	56.9 ± 6.5	0.671
tM (h)	0.001	0.038	1.000	0.929–1.080	0.980	82.0 ± 8.4	81.9 ± 9.4	0.981
tB (h)	−0.031	0.045	0.970	0.889–1.060	0.489	98.2 ± 5.5	100.0 ± 9.4	0.494
tPNf‐tPNa (h)	0.005	0.114	1.010	0.803–1.260	0.964	15.2 ± 2.4	15.1 ± 3.4	0.965
t2‐tPNf (h)	1.161	0.977	3.190	0.470–21.700	0.235	2.4 ± 0.4	2.3 ± 0.3	0.234
t3‐t2 (h)	−0.153	0.122	0.858	0.676–1.090	0.211	9.4 ± 3.9	10.8 ± 2.3	0.213
t4‐t3 (h)	0.085	0.115	1.090	0.868–1.360	0.462	2.2 ± 3.8	1.5 ± 2.1	0.903
t5‐t4 (h)	−0.006	0.068	0.994	0.870–1.140	0.935	11.5 ± 5.5	11.6 ± 4.4	0.891
t6‐t5 (h)	−0.064	0.095	0.938	0.778–1.130	0.503	1.9 ± 3.8	2.7 ± 3.6	0.206
t7‐t6 (h)	0.079	0.122	1.080	0.851–1.380	0.520	2.3 ± 3.8	1.7 ± 1.7	0.594
t8‐t7 (h)	−0.025	0.102	0.975	0.799–1.190	0.808	2.1 ± 3.9	2.4 ± 2.7	0.584
tM‐t8 (h)	0.017	0.035	1.020	0.949–1.090	0.632	26.6 ± 10.9	25.1 ± 8.2	0.642
tB‐tM (h)	−0.029	0.042	0.971	0.893–1.060	0.488	16.2 ± 7.6	18.1 ± 8.5	0.499
t3‐tPNf (h)	−0.134	0.117	0.875	0.696–1.100	0.253	11.8 ± 3.9	13.1 ± 2.4	0.378
t4‐tPNf (h)	−0.283	0.252	0.754	0.460–1.240	0.262	14.0 ± 1.0	14.5 ± 1.7	0.266
t5‐tPNf (h)	−0.024	0.063	0.976	0.863–1.110	0.706	25.5 ± 5.7	26.2 ± 5.1	0.727
t6‐tPNf (h)	−0.096	0.088	0.908	0.764–1.080	0.276	27.4 ± 4.0	28.8 ± 4.0	0.279
t7‐tPNf (h)	−0.044	0.079	0.956	0.823–1.110	0.563	29.7 ± 3.8	30.5 ± 5.1	0.715
t8‐tPNf (h)	−0.040	0.063	0.961	0.849–1.090	0.530	31.8 ± 5.3	32.9 ± 5.5	0.627
tM‐tPNf (h)	0.005	0.037	1.000	0.934–1.080	0.896	58.3 ± 8.2	58.0 ± 9.8	0.900
tB‐tPNf (h)	−0.030	0.048	0.971	0.884–1.070	0.535	74.6 ± 5.2	76.0 ± 8.6	0.541
ICM grade	0.539	0.670	1.710	0.461–6.370	0.421	–	–	–
TE grade	−0.921	0.579	0.398	0.128–1.240	0.112	–	–	–
Multivariate logistic analysis^b^								
Female age (years)	−0.209	0.113	0.792	0.641–0.979	0.031			
Migration speed of mNPBs (μm/h)	1.705	0.957	10.200	1.900–54.900	0.007			

Abbreviations: *β*, regression coefficient; CI, confidence interval; fNPBs, nucleolus precursor bodies in female pronuclei; h, hours; ICM, inner cell mass; mNPBs, nucleolus precursor bodies in male pronuclei; SE *β*, standard error of the regression coefficient; TE, trophectoderm; t2‐t8, first time of observation of the number of cells stated; tB, time of blastocyst formation (cavitation); tM, time of morula formation; tPNa, time of pronuclei appearance; tPNf, time of pronuclei disappearance.

^a^ Data are presented as mean ± standard deviation.

^b^ Relationship between ploidy status and all other factors was analyzed using univariate logistic analysis. Subsequently, multivariate logistic analysis was used to analyze the relationship between ploidy status and factors with *p* < 0.15 in univariate logistic analysis.

**FIGURE 3:**
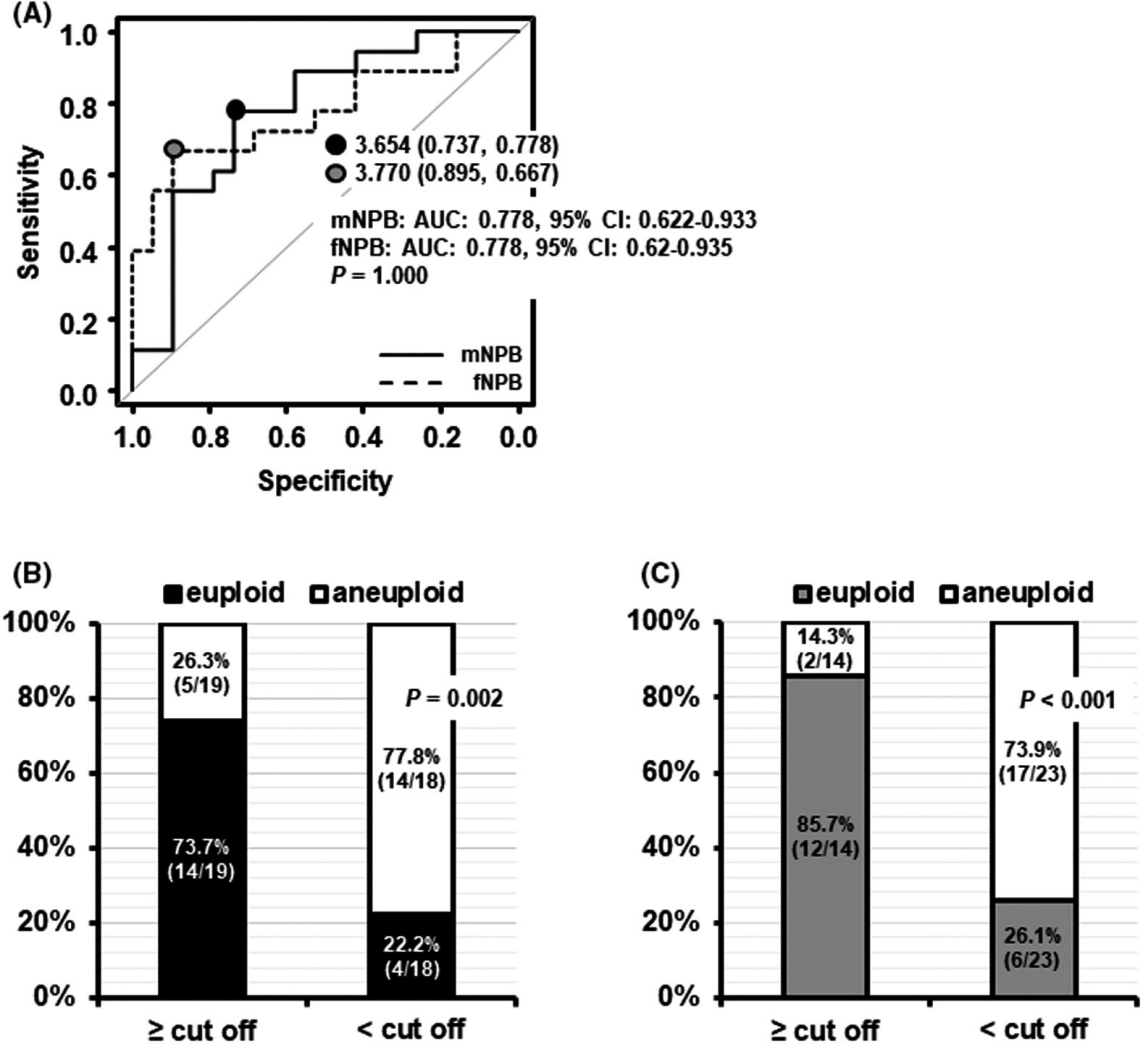
Evaluation of mNPB and fNPB migration speed as parameters for predicting ploidy status (euploid: *N* = 18; aneuploid: *N* = 19; 220 NPBs). (A) Comparing mNPB and fNPB migration speed as predictors of euploidy using a bootstrap test for 2 correlated ROC curves. (B) Ploidy status classified by the mNPB migration speed cut‐off value determined in ROC analysis. (C) Ploidy status classified by the cut‐off value for fNPB migration speed determined in ROC analysis. AUC, area under the curve; CI, confidence interval; fNPB, nucleolus precursor bodies in female pronuclei; mNPB, nucleolus precursor bodies in male pronuclei.

When the ability of mNPB migration speed to classify ploidy status was examined using ROC curve analysis, the cut‐off value was determined to be 3.65 μm/h (specificity, 73.7%; sensitivity, 77.8%; area under the curve [AUC], 0.78; 95% CI, 0.62–0.93) (Figure 3A). When the zygotes were categorized by this cut‐off value, the proportion of euploid zygotes with mNPB migration speeds ≥ the cut‐off value was significantly higher than in zygotes with mNPB migration speeds < the cut‐off value (73.7% vs. 22.2%, *p* = 0.002) (Figure 3B). When the ability of fNPB migration speed to classify euploidy was examined using ROC curve analysis, the cut‐off value was 3.77 μm/h (specificity, 89.5%; sensitivity, 66.7%; AUC, 0.78; 95% CI, 0.62–0.94, Figure 3A). When the zygotes were categorized by this cut‐off value, the proportion of euploid zygotes with fNPB migration speed ≥ the cut‐off value was significantly higher than that in zygotes with fNPB migration speed < the cut‐off value (85.7% vs. 26.1%, *p* < 0.001 Figure 3C). The accuracy to predict euploidy was not significantly different between the mNPB and fNPB migration speeds (*p* = 1.000, Figure 3A).

### 3.2 The migration speed of NPBs in the mPN is correlated with live birth after blastocyst transfer

**FIGURE 4:**
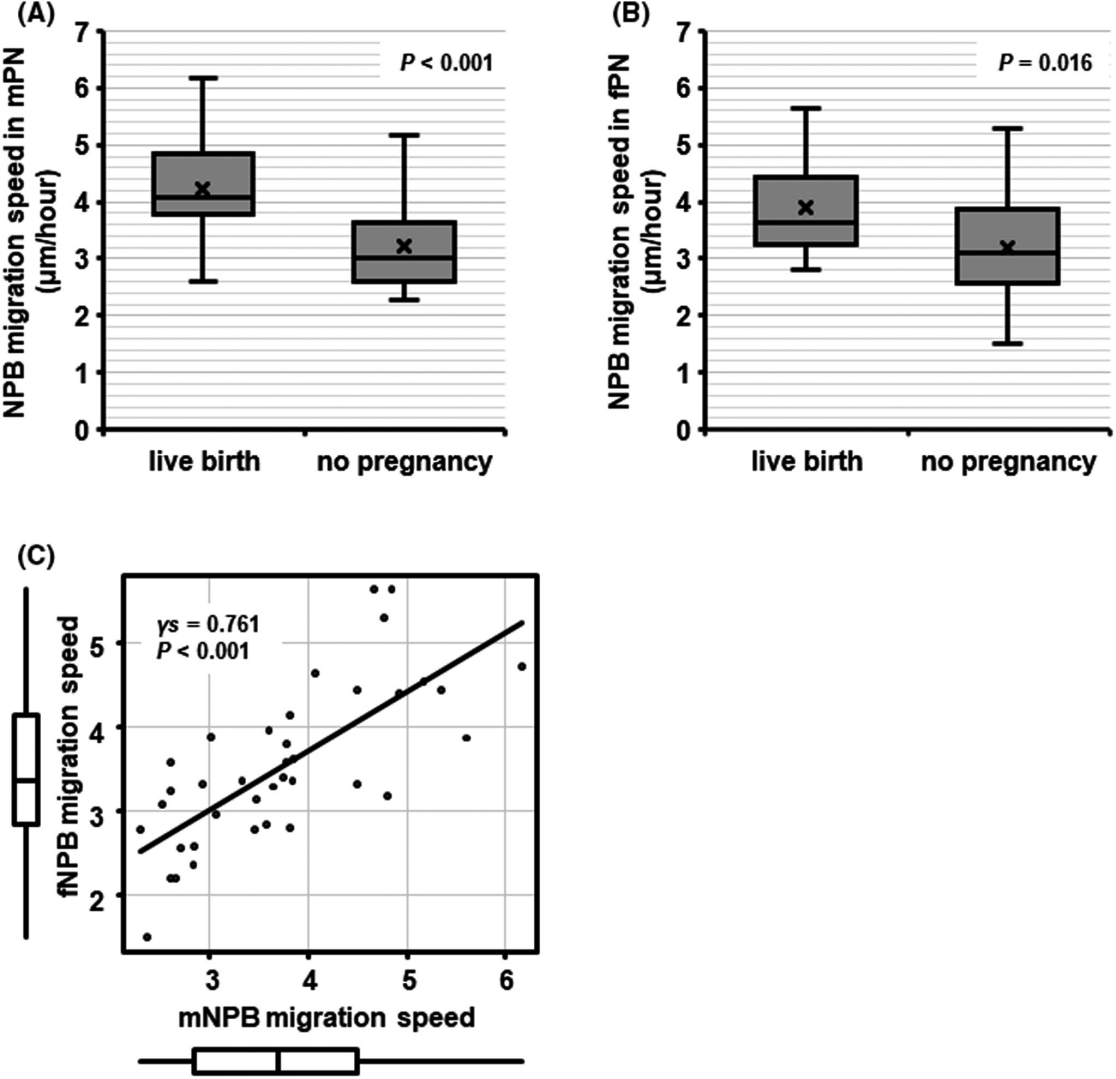
The relationship between migration speed of NPBs and live birth (live birth: *N* = 19; no pregnancy: *N* = 19; 225 NPBs). The migration speed of NPBs in (A) mPN and (B) fPN. The migration speeds of both mNPBs and fNPBs in patients with live birth were significantly faster than in patients with no pregnancy (mNPBs: 4.24 ± 0.93 vs. 3.23 ± 0.79 μm/h; fNPBs: 3.90 ± 0.84 vs. 3.18 ± 0.92 μm/h). (C) the relationship between the migration speed of mNPBs and the migration speed of fNPBs. fNPB, nucleolus precursor bodies in female pronuclei (fPN); mNPB, nucleolus precursor bodies in male pronuclei (mPN); γs, spearman correlation.

To test if the migration speed of NPBs was associated with LB, we conducted a retrospective analysis of patients with either LB or no pregnancy. The patients who gave LB were significantly older when OPU was performed than patients with no pregnancy (*p* = 0.014, Table 3). Blastocyst grades in patients with LB were significantly lower than in patients with no pregnancy (ICM: *p* = 0.019, TE: *p* = 0.002, Table 3). The migration speed was quantitated for 225 NPBs (114 mNPBs and 111 fNPBs) in 38 zygotes (3.00 ± 0.00 mNPBs/zygote and 2.92 ± 0.27 fNPBs/zygote). The migration speeds of both mNPBs and fNPBs in patients with LB were significantly faster than in patients with no pregnancy (mNPBs: 4.24 ± 0.93 vs. 3.23 ± 0.79 μm/h, *p* < 0.001, 1−*β* = 0.926; fNPBs: 3.90 ± 0.84 vs. 3.18 ± 0.92 μm/h, *p* = 0.016, 1−*β* = 0.672) (Figure 4A,B). Again, the migration speed of mNPBs was positively correlated with that of fNPBs (*γs* = 0.761, *p* < 0.001) (Figure 4C).

**TABLE 3:** Characteristics of blastocysts from vitrified‐warmed, single blastocyst transfer

	Live birth	No pregnancy	*p* value
No. of blastocysts	19	19	
Female (oocyte provider) age (years, mean ± SD)	34.2 ± 3.2	31.7 ± 2.7	0.014
Male (sperm provider) age (years, mean ± SD)	34.5 ± 4.4	32.6 ± 4.2	0.186
Controlled ovarian stimulation			
Long protocol	2 (10.5%)	0 (0%)	0.054
Short protocol	10 (52.6%)	13 (68.4%)	
Antagonist protocol	7 (36.8%)	6 (31.6%)	
Inner cell mass grade, *n* (%)			
Grade A	4 (21.1%)	9 (47.4%)	0.019
Grade B	9 (47.4%)	10 (52.6%)	
Grade C*	6 (31.6%)	0 (0%)	
Trophectoderm grade, *n* (%)			
Grade A	6 (31.6%)	5 (26.3%)	0.002
Grade B*	5 (26.3%)	14 (73.7%)	
Grade C*	8 (42.1%)	0 (0%)	

* *p*‐value <0.01 in the residual analysis following the chi‐square test.

**FIGURE 5:**
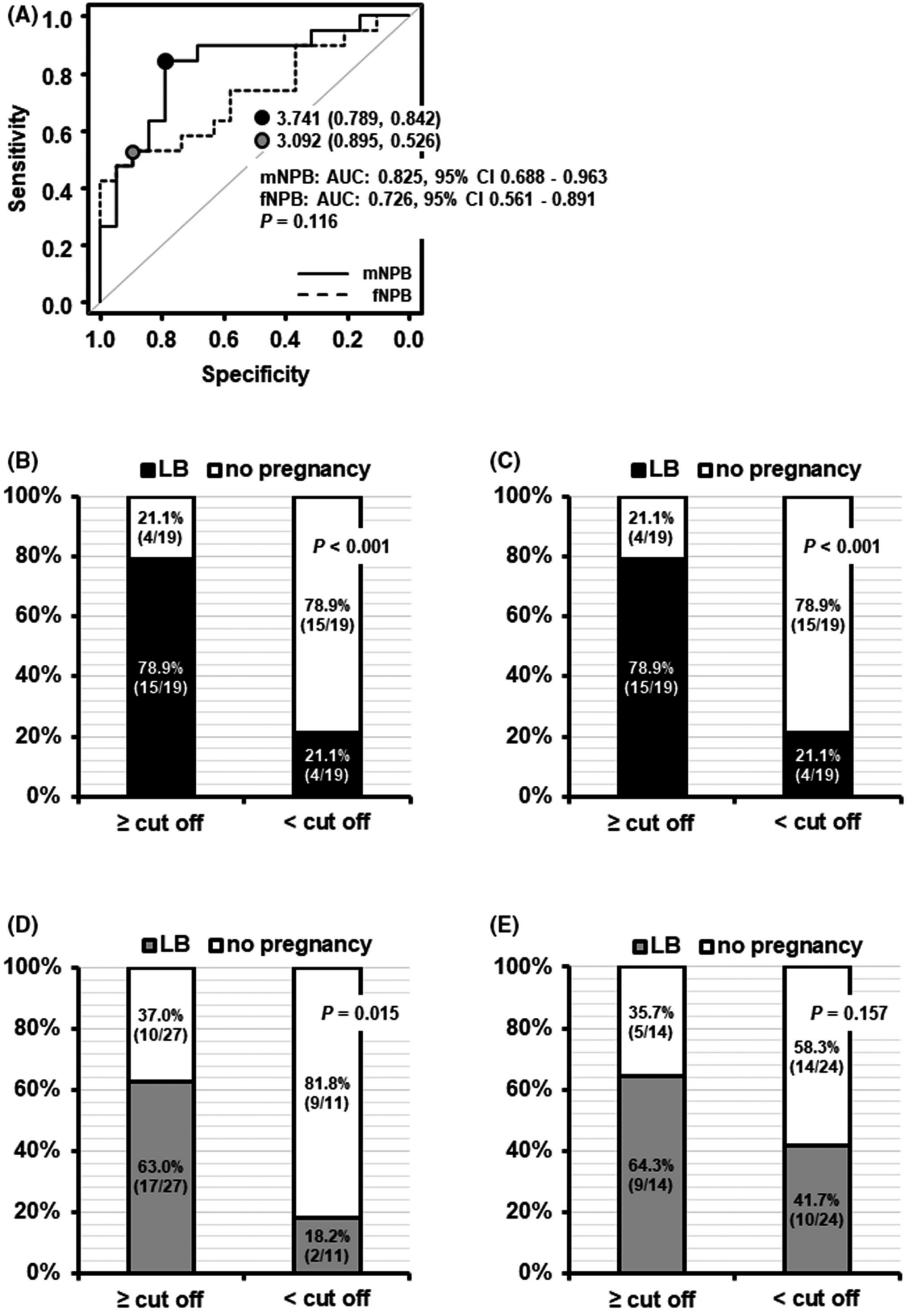
Evaluation of mNPB and fNPB migration speed as parameters for predicting live birth (live birth: *N* = 19; no pregnancy: *N* = 19; 225 NPBs). (A) Evaluation comparing mNPB migration speed and fNPB migration speed as predictors of live birth was completed using a bootstrap test for 2 correlated ROC curves. (B) the incidence of live birth when embryos were classified using the cut‐off value for mNPB migration speed determined in ROC analysis for live birth. (C) the incidence of live birth when embryos were classified using the cut‐off value for mNPB migration speed determined in ROC analysis for ploidy status. (D) the incidence of live birth when embryos were classified using the cut‐off value for fNPB migration speed determined in ROC analyses for live birth. (E) the incidence of live birth when embryos were classified using the cut‐off value for fNPB migration speed determined in ROC analysis for ploidy status. AUC, area under the curve; CI, confidence interval; fNPB, nucleolus precursor bodies in female pronuclei; mNPB, nucleolus precursor bodies in male pronuclei.

When a cut‐off value was used to examine the association between the migration speed of mNPBs and LB, the cut‐off value for the migration speed of the mNPBs was 3.74 μm/h (specificity, 78.9%; sensitivity, 84.2%; AUC: 0.825, 95% CI: 0.688–0.963, Figure 5A). When the zygotes were categorized using this cut‐off value, the proportion of LBs in zygotes with mNPB migration speed ≥ the cut‐off value was significantly higher than that in zygotes with mNPB migration speed < the cut‐off value. (78.9% vs. 21.1%, *p* < 0.001, Figure 5B). Moreover, when the zygotes were categorized using the cut‐off value determined for euploidy (3.65 μm/h), the proportion of LBs in zygotes with mNPB migration speed ≥ the cut‐off value was also significantly higher than that in zygotes with mNPB migration speed < the cut‐off value (78.9% vs. 21.1%, *p* < 0.001, Figure 5C).

When the ability of fNPB migration speed to classify LB was examined using ROC curve analysis, the cut‐off value was 3.09 μm/h (specificity, 89.5%; sensitivity, 52.6%; AUC: 0.726, 95%CI: 0.561–0.891, Figure 5A). The accuracy of the quantitative evaluation to predict LB was not significantly different for mNPB and fNPB migration speeds (*p* = 0.116, Figure 5A). When zygotes were categorized by the cut‐off value determined using fNPBs, the proportion of LBs in zygotes with fNPB migration speeds ≥ the cut‐off value was significantly higher than that in zygotes with fNPB migration speeds < the cut‐off value (63.0% vs. 18.2%, *p* = 0.015) (Figure 5D). However, when the zygotes were categorized by the cut‐off value for fNPB migration speed associated with ploidy status (3.77 μm/h), the proportion of LBs was not significantly different between zygotes with fNPB migration speeds ≥ the cut‐off value and zygotes with fNPB migration speeds < the cut‐off value (64.3% vs. 41.7%, *p* = 0.157) (Figure 5E).

Additionally, we investigated whether the accuracy of predicting LB could be improved by using cut‐off values for both mNPB and fNPB migration speed. The relationships between mNPB and fNPB migration speed in each zygote, cut‐off values, and LB were shown in Table 4. The proportion of LBs from zygotes with NPB migration speed ≥ the cut‐off values for both mNPBs and fNPBs was 77.8% (14/18), and the accuracy of prediction of LB was similar to the accuracy seen using cut‐off values for the migration speed of mNPBs only (78.9% [15/19]). Accordingly, these results suggest that the migration speed of mNPBs alone may be sufficient to predict LB after ART.

**TABLE 4:** The relationship between migration speed of the NPBs in each zygote, cut‐off values, and live birth

Patient ID	Migration speed of mNPBs (μm/h)^a^		Migration speed of fNPBs (μm/h)^b^		Live birth or no pregnancy
1	4.799	≥ cut‐off	3.188	≥ cut‐off	Live birth
2	4.842	≥ cut‐off	5.623	≥ cut‐off	Live birth
3	3.804	≥ cut‐off	4.134	≥ cut‐off	No pregnancy
4	3.778	≥ cut‐off	3.814	≥ cut‐off	Live birth
5	2.604	<	3.242	≥ cut‐off	Live birth
6	4.655	≥ cut‐off	5.635	≥ cut‐off	Live birth
7	3.741	≥ cut‐off	3.409	≥ cut‐off	No pregnancy
8	2.831	<	2.377	<	No pregnancy
9	4.489	≥ cut‐off	4.437	≥ cut‐off	Live birth
10	2.653	<	2.211	<	No pregnancy
11	3.813	≥ cut‐off	2.806	<	Live birth
12	3.855	≥ cut‐off	3.631	≥ cut‐off	Live birth
13	2.280	<	2.791	<	No pregnancy
14	3.469	<	3.147	≥ cut‐off	Live birth
15	3.052	<	2.958	<	Live birth
16	2.836	<	2.581	<	No pregnancy
17	3.570	<	2.851	<	No pregnancy
18	2.925	<	3.318	≥ cut‐off	Live birth
19	4.770	≥ cut‐off	5.301	≥ cut‐off	No pregnancy
20	3.010	<	3.882	≥ cut‐off	No pregnancy
21	6.165	≥ cut‐off	4.720	≥ cut‐off	Live birth
22	3.323	<	3.356	≥ cut‐off	No pregnancy
23	2.592	<	3.586	≥ cut‐off	No pregnancy
24	2.512	<	3.092	≥ cut‐off	No pregnancy
25	5.348	≥ cut‐off	4.440	≥ cut‐off	Live birth
26	4.073	≥ cut‐off	4.640	≥ cut‐off	Live birth
27	2.365	<	1.512	<	No pregnancy
28	3.455	<	2.780	<	No pregnancy
29	4.925	≥ cut‐off	4.400	≥ cut‐off	Live birth
30	3.645	<	3.298	≥ cut‐off	No pregnancy
31	3.775	≥ cut‐off	3.592	≥ cut‐off	Live birth
32	3.599	<	3.962	≥ cut‐off	No pregnancy
33	2.694	<	2.567	<	No pregnancy
34	3.839	≥ cut‐off	3.372	≥ cut‐off	Live birth
35	2.601	<	2.211	<	No pregnancy
36	5.605	≥ cut‐off	3.875	≥ cut‐off	Live birth
37	4.487	≥ cut‐off	3.331	≥ cut‐off	Live birth
38	5.168	≥ cut‐off	4.537	≥ cut‐off	No pregnancy

Abbreviations: fNPBs, nucleolus precursor bodies in female pronuclei; mNPBs, nucleolus precursor bodies in male pronuclei.

^a^ Cut‐off value for live birth calculated for the migration speed of mNPBs: 3.741 μm/h.

^b^ Cut‐off value for live birth calculated for the migration speed of fNPBs: 3.092 μm/h.

### 3.3 The relationship between migration speed of NPBs in the mPN and miscarriage

**FIGURE 6:**
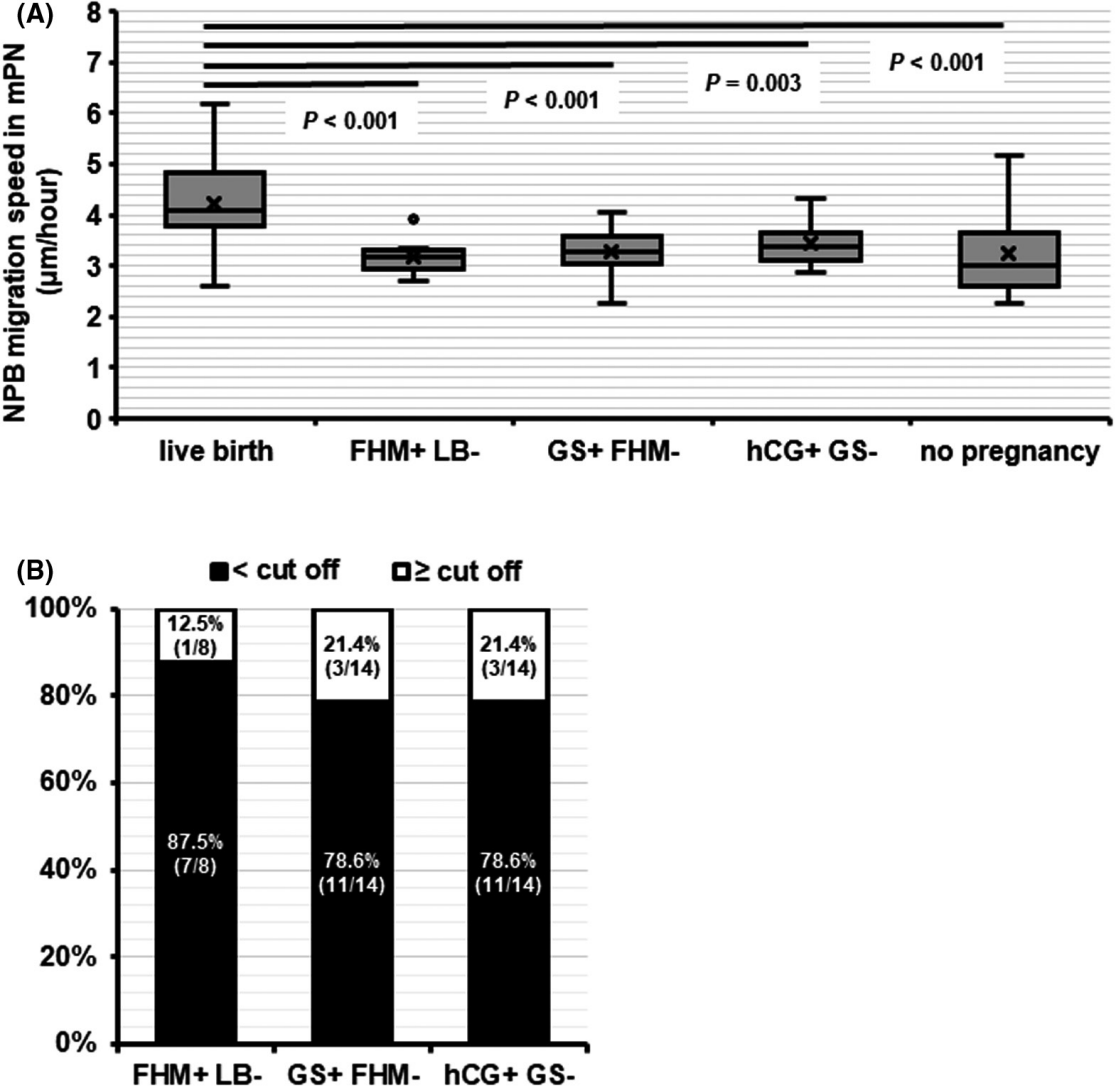
The relationship between migration speed of NPBs in mPN and miscarriage or biochemical pregnancy (FHM+/LB−: *N* = 8, GS+/FHM−: *N* = 14, hCG+/GS −: *N* = 14, LB: *N* = 19, no pregnancy: *N* = 19). The migration speed of NPBs in (A) mPN. The migration speeds of mNPBs in patients with miscarriages or biochemical pregnancy were significantly slower than in patients with LB (FHM+/LB−; 3.19 ± 0.36, GS+/FHM−; 3.27 ± 0.45, hCG+/GS−; 3.44 ± 0.45, vs. LB; 4.24 ± 0.93 μm/h). (B) Classification of miscarried zygotes using the cut‐off value for mNPB migration speed determined in ROC analysis for ploidy status. FHM, fetal heart movement; GS, gestational sac; hCG, human chorionic gonadotropin; LB, live birth; mPN, male pronuclei; NPB, nucleolus precursor body.

To better understand the relationship between mNPB migration speed and miscarriage, we conducted a retrospective analysis of patients with miscarriages (FHM+/LB− or GS+/FHM−) or biochemical pregnancy (hCG+/GS−). The age of the women with GS+/FHM− miscarriages was significantly higher than that of women with LB or no pregnancy (*p* < 0.05, Table 5). The migration speed was quantitated for 108 mNPBs in 36 zygotes from patients with miscarriage (3.00 ± 0.00 mNPBs/zygote) and compared with previously determined migration speeds in patients with LB (Figure 4A). The migration speeds of mNPBs in patients with miscarriages or biochemical pregnancy were significantly slower than in patients with LB (FHM+/LB− 3.19 ± 0.36 μm/h, GS+/FHM− 3.27 ± 0.45 μm/h, hCG +/GS− 3.44 ± 0.45 μm/h, vs. LB 4.24 ± 0.93 μm/h, *p* ≤ 0.003) (Figure 6A). The migration speed of the mNPBs was not significantly different between miscarriages, biochemical pregnancy, and no pregnancy (Figure 6A). When the zygotes were categorized using the cut‐off value determined for euploidy (3.65 μm/h), the proportion of FHM+/LB−, GS+/FHM−, and hCG+/GS− zygotes with mNPB migration speed < the cut‐off value was very high (87.5% [7/8], 78.6% [11/14], and 78.6% [11/14], respectively; Figure 6B).

**TABLE 5:** Characteristics of blastocysts/embryos with miscarriage after transfer

	Live birth	FHM+/LB−	GS+/FHM−	hCG+/GS−	No pregnancy
No. of patients	19	8	14	14	19
Female (oocyte provider) age (years, mean ± SD)	34.2 ± 3.2	34.8 ± 5.7	36.8 ± 4.6*,**	33.5 ± 4.0	31.7 ± 2.7
No. of good quality blastocysts	10	4	7	7	19
No. of poor‐quality blastocysts	9	2	6	7	0
No. of early embryos (≥7 cell‐embryo)	0	2	1	0	0

Abbreviations: FHM, fetal heart movement; GS, gestational sac; hCG, human chorionic gonadotropin.

* *p*‐value <0.05 in Mann–Whitney *U* test (vs. live birth).

** *p*‐value <0.01 in Mann–Whitney *U* test (vs. no pregnancy).

### 3.4 The relationship between the migration speed of NPBs and clinical outcome after ART when the oocyte provider was ≥40 years old

**FIGURE 7:**
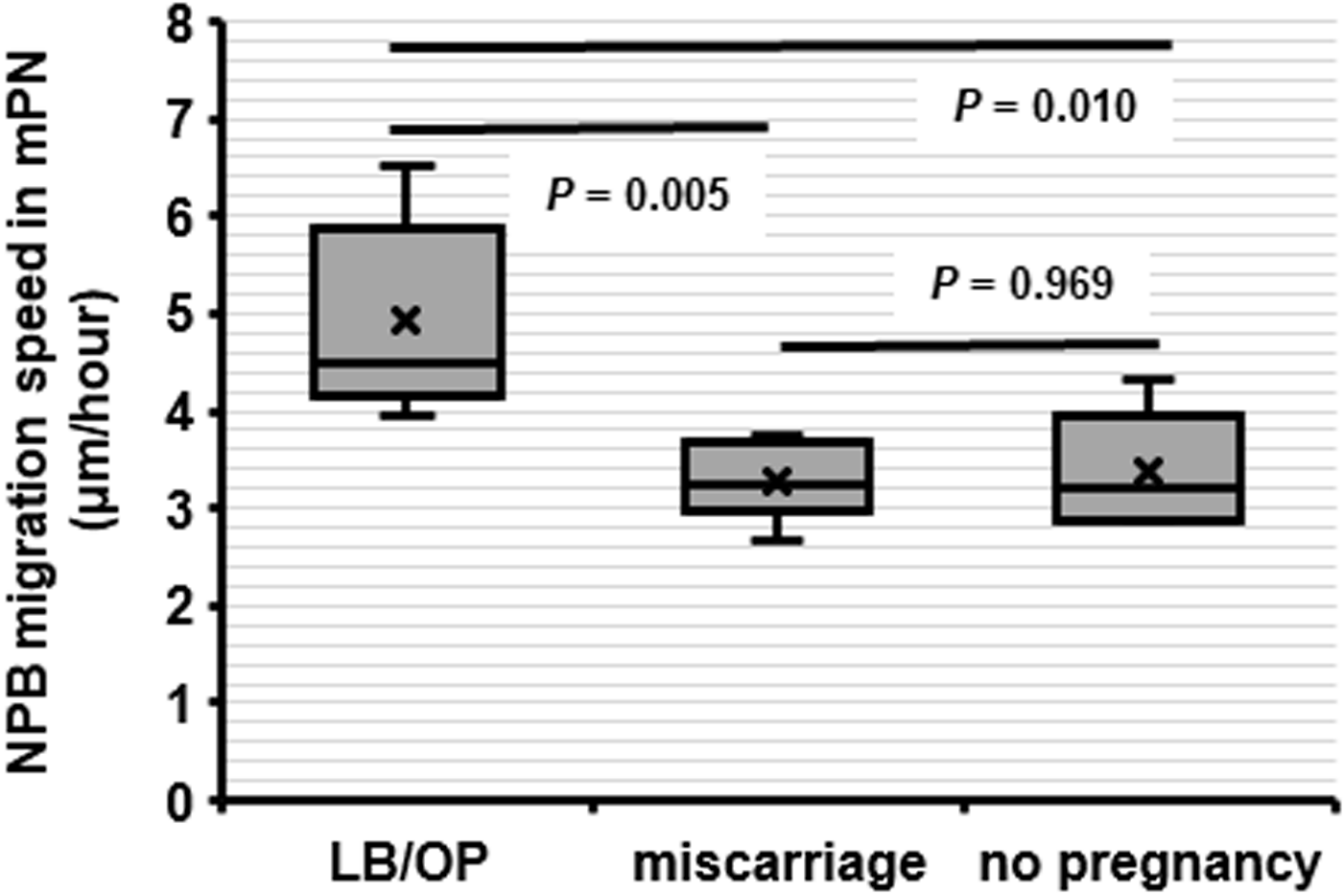
The relationship between migration speed of NPBs in mPN and clinical outcome after ART in patients (oocyte providers) ≥40 years old (LB/OP: *N* = 5, miscarriage: *N* = 6, no pregnancy: *N* = 5). The migration speeds of mNPBs in patients with LB/OP were significantly faster than in patients with miscarriage and no pregnancy (4.91 ± 1.01 vs. 3.27 ± 0.40 vs. 3.37 ± 0.60 μm/h). LB/OP, live birth, or ongoing pregnancy; mPN, male pronuclei; NPB, nucleolus precursor body.

Finally, we investigated the relationship between mNPB migration speed and clinical outcome after ART when the oocyte provider was ≥40 years of age. The women's ages were not different between the LB/OP, miscarriage, and no pregnancy study groups (Table 6). The migration speed was quantitated for 48 mNPBs in 16 zygotes from females ≥40 years of age (3.00 ± 0.00 mNPBs/zygote). The migration speeds of mNPBs in patients with LB/OP were significantly faster than in patients with miscarriage or no pregnancy (4.91 ± 1.01 vs. 3.27 ± 0.40 vs. 3.37 ± 0.60 μm/h, *p* ≤ 0.01) (Figure 7). Migration speeds were similar to those observed in relatively young patients (Figures 6A and 7).

**TABLE 6:** Characteristics of embryos and blastocysts from female patients ≥40 years old

	LB/OP	Miscarriage	no pregnancy
No. of patients	5	6	5
Female (oocyte provider) age (years, mean ± SD)	41.0 ± 1.0	40.8 ± 0.8	41.4 ± 1.3
No. of good quality blastocysts	1	3	2
No. of poor‐quality blastocysts	3	2	2
No. of early embryos (≥7‐cell embryo)	1	1	1

Abbreviation: LB/OP, live birth or ongoing pregnancy.

## 4 DISCUSSION

This study convincingly demonstrated that the migration speed of NPBs in IVF‐derived zygotes is associated with ploidy and LB. The NPBs in IVF‐derived euploid zygotes migrated faster than those in aneuploid zygotes. The NPBs in zygotes having the potential to reach LB migrated faster than those in zygotes that did not result in a sustainable pregnancy. This is in agreement with similar findings from our previous study in ICSI‐derived zygotes.^11^ Accordingly, the migration speed of NPBs, specifically that of mNPBs, may be a useful predictor of LB after ART regardless of the insemination method employed. Additionally, a cut‐off value for mNPB migration speed that predicted LB and euploidy identified in this study may be useful for selecting zygotes with the potential to develop into a baby. Our results suggest that evaluation of mNPB migration speed has clinical value for embryo selection, and consequently, the time to LB after the initiation of fertility treatments could be shortened using this analysis.

In this study, the ploidy status of a zygote was associated with the patient's age at OPU and the migration speed of mNPBs in the zygote in a multivariate analysis. The association of ploidy status with maternal age is well established,^26^, ^27^, ^28^ but the latter finding is novel for IVF‐derived zygotes. The predictive value of mNPB migration speed was larger than the predictive value of fNPB migration speed when examining euploidy and LB both in IVF‐derived zygotes and in ICSI‐derived zygotes.^11^ The paternal genome undergoes protamine‐histone exchange immediately after fertilization and is actively demethylated in the zygote. In contrast, the maternal genome undergoes passive demethylation via DNA replication during cleavage.^29^, ^30^ Hence, transcriptional activity is higher in the mPN than in the fPN in the zygote.^31^ We speculate that this difference in transcriptional activity affects the predictive value of NPB migration speed for euploidy and LB. Moreover, we previously found that although 96% of NPBs were available for tracking, approximately 1.6‐fold more mNPBs were available than fNPBs.^11^ Accordingly, we believe that evaluation of mNPBs may be more useful clinically than evaluation of fNPBs.

Because NPBs co‐localize with chromatin, NPB position can serve as a proxy for chromatin distribution in the zygote.^7^, ^8^ Migration of chromatin in the nucleus has been reported in eukaryotes, such as yeast^32^ and Drosophila,^33^ and in human cell lines.^34^, ^35^, ^36^ Fast and slow chromatin movements are affected by nucleosome movement in human cells.^36^ In mammals, no change in the mobility of chromosomal regions occurs between cells in mid‐G1‐, late G1‐, S‐, and G2‐phase nuclei, whereas in early G1‐phase nuclei, the chromosomes are significantly more mobile as compared with later stages of the cell cycle.^37^ Moreover, an increase in transcriptional output may enhance the mobility of a locus to facilitate its re‐localization to the appropriate nuclear compartment.^37^ Restriction of DNA movement in the nucleus is connected to the levels of transcriptional activity of the RNA polymerase II (RNAPII) associated with the chromatin.^38^ Nagashima and colleagues showed that active RNAPII globally constrains chromatin movements, and RNAPII inhibition or its rapid depletion releases the chromatin constraints and increases chromatin dynamics.^39^ During mid‐ and late G1 phase, mRNA transcription occurs with no change in the mobility of chromosomal regions.^37^ Because the movement of the NPBs indirectly indicates chromatin movement, we speculated that aggressive movement of the NPBs indicates termination of a smooth transcription process, which promotes euploidy and LB.

Chromosomal aneuploidy in human embryos is acquired during mitosis.^40^ The nucleolar protein complexes are required for mitotic chromosome segregation and crucial for the stability of mitotic chromosome segregation.^41^ Nucleoplasmin 2, a nucleolar protein, reconstitutes NPBs and is required for centromeric/pericentromeric chromatin organization.^42^ Additionally, the nucleoplasmin 2‐based oocyte‐nucleolus structure is an essential platform for organization of pericentromeric heterochromatin for correct segregation of the chromosomes.^42^ Accordingly, structural abnormalities in NPBs may result in chromosome segregation abnormalities, which lead to chromosomal abnormalities. Hence, we speculated that the migration speed of NPBs would reflect whether the structure of the NPBs is normal or abnormal. This study showed that the migration speed of NPBs in aneuploid zygotes was slower than in euploid zygotes. Moreover, NPB migration in patients with miscarriage or no pregnancy was slower than that of patients with LB. Therefore, we postulate that the migration speed of NPBs indicates structural normality or abnormality of the NPBs and indirectly signifies the ploidy status.

In this study, the differences in the fertilization process between ICSI and IVF, such as several biological barriers of gamete selection,^12^ inseminated sperm structures,^13^ oocyte activation,^14^ and DNA replication,^15^ did not affect NPB migration speed or the ability of NPB migration speed to predict LB. This may be because NPBs are maternally inherited and originate from material that is present in oocyte germinal vesicle and reappears in both PNs.^6^ The ability of NPB migration speed to predict outcomes after either procedure is supported by the observation that morphological events during the fertilization process after extrusion of the second polar body and their timing are not different between ICSI and IVF.^9^

This study demonstrated that NPBs migrate significantly faster in IVF‐derived zygotes of patients who go on to have a LB than in patients with no pregnancy. When a cut‐off value was determined and applied, the proportion of LBs was 78.9%. Our previous study showed that when a cut‐off value for NPB migration speed in an ICSI‐derived blastocyst that predicted LB was determined and applied, the proportion of LB/OP was 75%.^11^ Single euploid VBT cycles have yielded LB rates of ~60%.^43^, ^44^ Therefore, we believe that analysis of NPB speed may be comparable with PGT‐A in predicting the viability of the zygote regardless of the insemination method used.

As we described in a previous study,^11^ there are some difficulties encountered when analyzing NPB migration speed, such as when the mPN overlaps with fPN, so the technique has some disadvantages. Nonetheless, analysis of NPB migration speed may be an improvement over PGT‐A for assessing embryos prior to embryo transfer. PGT‐A is expensive, invasive, and carries risks, and it is difficult to perform a biopsy when there are few TE cells. Non‐invasive PGT‐A, which is performed using cell‐free DNA from the embryo culture medium and the blastocoel fluid,^45^ carries a risk of genetic contamination.^46^, ^47^ Analysis of NPB migration speed may circumvent these problems. We believe that analysis of mNPB movement could become an alternative, non‐invasive method for blastocyst selection prior to transfer that would be performed prior to or instead of PGT‐A. A large, prospective study and/or multi‐institutional collaborative research is necessary to determine the clinical utility of NPB‐migration‐speed screening and its place among established screening methods. Further work is also needed to mechanistically explain the relationship between NPB migration and embryo development.

This study has some limitation. First, NPB migration along the *z*‐axis could not be analyzed. Second, NPB tracking could not be performed when there were many NPBs or the NPBs moved drastically. Third, the number of patients >40 years of age at OPU with miscarriage or LB/OP was small, in part because transfers in some patients with recurrent ART failure comprised 2 blastocysts, 2 embryos, or an embryo and a blastocyst. However, the results in women (oocyte providers) >40 years old were similar to the results of relatively young patients, so evaluation of mNPB migration speed in zygotes from women older than 40 years of age has potential clinical value for embryo selection. Moreover, we could not investigate the relationship between the migration speed of NPBs and clinical outcomes after fresh embryo transfer because our clinic uses a freeze‐all strategy. Several studies have demonstrated that LB rates are not significantly affected by the freeze‐all strategy.^48^, ^49^ Accordingly, we anticipate that the freezing process did not negatively affect the relationships we observed.

## 5 CONCLUSION

This study, when coupled with our previous work,^11^ demonstrates that zygotes with fast NPB migration speed possess the developmental potential for LB regardless of the insemination method used to generate them. The migration speed of mNPBs is a novel predictor of ploidy status and LB. The migration speed of mNPBs may have clinical value for embryo selection and is an attractive marker for non‐invasive human embryo selection.

## CONFLICT OF INTEREST

The authors declare no conflicts of interest related to this study.

## ETHICS STATEMENT

All procedures followed were in accordance with the ethical standards of the responsible committee on human experimentation (institutional and national) and with the Helsinki Declaration of 1964 and its later amendments. Informed consent was obtained from all patients for being included in the study. This study was approved by the Umeda Fertility Clinic Institutional Review Board (181215R1).
